# Supplementary material for: A Novel Carbohydrate Fatty-Acid Monosulphate Ester, Squalane-in-Water Adjuvant Is Safe and Enhances Inactivated Influenza Vaccine Immunogenicity in Older Adults
Source: Vaccines (Basel). 2025 Aug 29;13(9):922. doi: 10.3390/vaccines13090922 (PMC12474436; doi:10.3390/vaccines13090922)
Supplement: Supplementary file 1 [file vaccines-13-00922-s001.zip › vaccines-3814957-supplementary.pdf]

Supplementary information

A novel carbohydrate fatty acid monosulphate squalane-in-water adjuvant is safe and boosts inactivated influenza vaccine immunogenicity in older adults

Valentino D'Onofrio[1], Bart Jacobs[1], Azhar Alhatemi[1], Simon De Gussem[1], Marjolein Verstraete[1], Sharon Porrez[1], Anthony Willems[1], Fien De Boever[1], Gwenn Waerlop[1], Geert Leroux-Roels[1], Els Michels[2], Francesca Vanni[3], Alessandro Manenti[3], Peter Paul Platenburg[4], Luuk Hilgers[4], Isabel Leroux-Roels[1]

Affiliations

[1] Center for Vaccinology (CEVAC), Ghent University and Ghent University Hospital, Ghent, Belgium

[2] Harmony Clinical Research BV, Melle, Belgium

[3] VisMederi S.r.l., Monteriggioni, Italy

[4] LiteVax, Ophemert, The Netherlands

Corresponding author:

Isabel Leroux-Roels, MD, PhD

Center for Vaccinology, Ghent University and Ghent University Hospital

Corneel Heymanslaan 10, 9000 Ghent, Belgium

isabel.lerouxroels@uzgent.be

Supplementary methods

Humoral immune response

Five mL of blood were collected by venous puncture in serum separation blood collection tubes (Becton Dickinson Vacutainer tubes) from all participants at baseline (Day 0) and at Day 7, Day 28 and Day 180. Serum was collected after centrifugation for 10 minutes at 1300-2000g and frozen at 400µL per aliquot.

Humoral immune response was evaluated through Haemagglutination Inhibition (HI) Assay and live virus Micro-Neutralization assay using an ELISA-based read out (MNE). In the HI Assay, all serum samples were pre-treated with receptor destroying enzyme (RDE) (ratio 1:5) from Vibrio Cholerae (Sigma-Aldrich) for 18 h at 37° C in a water bath, then heat inactivated for 1 h at 56° C in a water bath with 8% sodium citrate (ratio 1:4). Serum samples were pre-diluted 1:10 with 0.9% saline solution, then in a 96-well plate, 2-fold serially diluted in ten consequent wells. Each serum sample was tested in duplicate. Twenty-five microliters of standardized antigen (A/Victoria (H1N1) 20/232, A/Darwin (H3N2) 21/318, B/Phuket 21/136 and B/Austria 21/316 all provided by NIBSC), were added to each well and the mixture was incubated at room temperature for one hour. Turkey Red Blood Cells (RBCs) were centrifuged two times, washed with 0.9% saline solution, and adjusted to a final dilution of 0.35%. Fifty microliters of RBCs were added in each well and, after one hour of incubation at room temperature, the plates were visually evaluated for the presence of agglutination inhibition. The antibody titre was expressed as the reciprocal of the highest serum dilution showing complete inhibition of agglutination.

For the MNE assay, heat inactivated serum samples were pre-diluted 1:10, then 2-fold serially diluted in ten consequent wells in a 96-well plate. A standardized amount (100 tissue culture infective dose 50% (TCID50)) of live A/Victoria (H1N1), A/Darwin (H3N2), B/Phuket or B/Austria influenza virus (provided by NIBSC) was added to each well and mixed, then plates were incubated for 1 hour at 37 °C and 5% CO2 in a humidified atmosphere. After the incubation period, 1.5x103 MDCK cells (ATCC) were added in each well to the virus-serum mixture. The plates were then incubated for 16-20 hours at 37°C and 5% CO2 in humidified atmosphere. After overnight incubation, wells were washed, and cells were fixed using cold fixative acetone for 10-12 minutes. Primary antibody against virus N protein was diluted and added to each well: Anti-Influenza A Antibody, nucleoprotein, clone A1 (Millipore) was used for A/Victoria (H1N1) and A/Darwin (H3N2); Mouse Anti Influenza B Monoclonal Antibody (Bio-Rad) was used for B/Phuket and B/Austria. Wells were washed and goat anti-mouse IgG HRP-conjugated

secondary antibody (Sigma-Aldrich) was used. Substrate solution was prepared and added to plates and incubated 10 minutes RT to develop a colorimetric reaction. Stop solution was added and plate optical densities (ODs) were immediately evaluated using an automatic ELISA reader equipped with 490nm wavelength. Each plate had four Cell Control (CC) wells showing no infection, and four Viral Control (VC) wells. Average ODs from CC and VC were used to calculate the infection cut-off value, namely the OD value at which 50% MDCK cells were infected. Based on this, the reciprocal of the highest serum dilution corresponding to the 50% of protection against virus infection represents the neutralization antibody titre for the tested sample.

Both in HI and MNE assays, a positive control having high antibody response for the homologous strain (Sheep antisera A/Victoria (H1N1) 21/120, A/Darwin (H3N2) 21/324, B/Phuket 19/322 and B/Austria 21/326, all provided by NIBSC) and a negative control were used as assays internal quality controls.

Influenza surveillance

Material for nasal self-testing was provided to the participants on the day of vaccination. The nasal self-swab was able to detect influenza and COVID-19. Participants were instructed to contact the site promptly once they experience simultaneously at least one respiratory symptom and at least one systemic symptom.

Respiratory symptoms included:

- Nasal congestion (‘Runny or dripping nose’, ‘Congested or stuffy nose’, ‘Sinus pressure’)
- Sore throat (‘Scratchy of itchy throat’, ‘Sore or painful throat’, ‘Swollen throat’, ‘Difficulty swallowing’)
- Conjunctivitis or eye irritation (‘Teary or watery eyes’, ‘Sore or painful eyes’, ‘Eyes sensitive to light’)
- Dyspnea, wheezing or cough (‘Trouble breathing’, ‘Chest congestion’, ‘Chest tightness’, ‘Dryor hacking cough’, ‘Wet or loose cough’, ‘Coughing’, ‘Coughed up mucus or phlegm’)

Systemic symptoms included:

- Fever ≥37.5°C (measured oral temperature), or feeling feverish (‘Chills or shivering, ‘‘Felt cold’, ‘Felt hot’, ‘Sweating’)
- Fatigue (‘Sleeping more than usual’, feeling ‘Weak or tired’)
- Joint or muscle pain (‘Body aches or pains’)
- Malaise or general discomfort (‘Felt dizzy’, ‘Felt lightheaded’, ‘Felt nauseous’, ‘Lack of appetite’, ‘Stomach ache’, ‘Felt uncomfortable (general discomfort)’, ‘Difficulty staying asleep’, ‘Difficulty falling asleep’)
- Headache (‘Head congestion’, ‘Headache’)

Participant were instructed to perform a nasal swab at home and to contact the study team again to inform them about the outcome of the self-swab. New sampling kits for nasal self-testing were distributed at every visit in case the participant would already have used or lost the ones given at the previous visit. Participants were trained at the vaccination visit on how to correctly use the nasal self-swab. Written instructions were provided to the participants on the vaccination visit.

Supplementary Table 1. In- and exclusion criteria.

Inclusion criteria	
1	Written signed informed consent obtained before any study-related activities.
2	Aged 18 to 50 years inclusive, at the time of signing the ICF in the younger age cohorts and aged 60 years or older, at the time of signing the ICF in the older age cohorts.
3	Participants who are considered to be in good general health as determined by medical evaluation including medical history, physical examination and laboratory tests within 21 days prior to enrollment.
4	Participants with a BMI within the range 18.5 to 35 kg/m2 inclusive at screening.
5	Women who are not pregnant or breastfeeding, and one of the following conditions applies:<br>• Women of non-childbearing potential (WONCBP).<br>Non-childbearing potential is defined as surgically sterilized (e.g. hysterectomy, bilateral oophorectomy, or tubal ligation/salpingectomy) or postmenopausal (defined as having no menstrual bleeding for at least 12 months) without an alternative medical cause prior to study.<br>OR<br>• WOCBP and using a highly effective contraceptive method (with a failure rate of less than 1 % per year) from at least 1 month prior to study vaccination and for 3 months post-vaccination.<br>The investigator should evaluate the potential for contraceptive method failure (e.g., noncompliance, recently initiated) in relationship to study vaccination. Highly effective contraception is defined as stabilized on oral birth control for at least 1 month before study participation, intrauterine device/system, implant, injection, transdermal patch, vasectomized partner, or sexual abstinence (excluding periodic abstinence). The reliability of sexual abstinence needs to be evaluated in relation to the duration of the study and the preferred and usual lifestyle of the participant. The participant should commit her abstinence to at least 1 month prior to study vaccination and for 3 months post-vaccination. If the participant will not maintain abstinence and changes her status, the participant must first commit to another highly effective method of contraception, which should be discussed with the investigator prior to terminating sexual abstinence as contraceptive method.
6	WOCBP must have a negative serum pregnancy test at screening and a negative urine pregnancy test before vaccination at Day 1. The investigator is responsible for review of medical history and menstrual history to decrease the risk for inclusion of a woman with an early undetected pregnancy.
7	Participants who are willing and able to comply with the study procedures and are in the view of the investigator capable of completing the study.
Exclusion criteria	
1	History of previous laboratory confirmed influenza infection in the past 6 months, excluding laboratory confirmed COVID-19 infections, prior to the day of study vaccination.
2	Positive (in the past, suspected or ongoing) for hepatitis B surface antigen (HBsAg), hepatitis C virus (HCV) antibody, and human immunodeficiency virus (HIV) antibody.
3	Past or current history of immune mediated and/or autoimmune diseases as indicated by the investigator, e.g., diabetes mellitus type I and thyroid disease.

4	Serious reactions to vaccines that preclude receipt of study vaccinations as determined by the investigator.
5	Clinical conditions representing a contraindication for IM administration, as judged by the investigator, e.g., history of bleeding disorder (e.g., factor deficiency, coagulopathy, or platelet disorder requiring special precautions) or significant bruising or bleeding difficulties with IM administration or blood draws.
6	History of confirmed hypersensitivity, allergy and/or anaphylaxis to eggs (ovalbumin or chicken proteins), squalene-based adjuvants, or other components of the study vaccine (neomycin, formaldehyde, or octoxinol-9).
7	Current history of uncontrolled medical illness (unstable for the past 3 months) as indicated by investigator, e.g., hypertension, diabetes mellitus type II.
8	Past or current history of any neurological disorder, e.g., Guillain-Barré syndrome and seizure disorder other than: 1) febrile seizures, 2) seizures secondary to alcohol withdrawal more than 3 years ago, or 3) seizures that have not required treatment within the last 3 years.
9	History of asplenia, functional asplenia or any condition resulting in the absence or removal of the spleen.
10	Active malignancy or malignancy within the past 5 years, except basocellular carcinoma (single lesion) that has been fully removed.
11	Asthma that is unstable or required emergent care, urgent care, hospitalization or intubation during the past two years or that is expected to require the use of oral or intravenous corticosteroids.
12	History of hereditary angioedema (HAE), acquired angioedema (AAE) or idiopathic forms of angioedema.
13	History of idiopathic urticaria within the past year.
14	Current or recent (< 2 years ago) heavy smoking (> 20 cigarettes per day). If candidate stopped smoking > 2 years ago, the investigator will make an individual judgement based on the total packs per year and the candidate's overall health status. Drug - or alcohol abuse/addiction (including alcohol dependence), or psychiatric condition (e.g. past or present psychoses; disorder requiring lithium; or within 5 years prior to administration of study vaccine, a history of suicide plan or attempt), which in the investigator's opinion could compromise the participant's safety and/or compliance with the protocol.
15	A rash, dermatological condition or tattoos that would, in the opinion of the investigator, interfere with injection local reaction rating.
16	Prior receipt of an investigational or licensed seasonal or pandemic influenza vaccine in the 9 months before administration of study vaccine or planning to receive the influenza vaccination during the study period.
17	Prior receipt of a live attenuated vaccine in the 28 days prior to administration of study vaccine, or within 14 days for subunit or inactivated vaccines other than seasonal or pandemic influenza vaccination, excluding COVID-19 vaccine.
18	Prior receipt of vaccination with TETRALITE + 0.5 mg LVA or TETRALITE + 2 mg LVA.
19	Prior receipt of COVID-19 vaccine in the 7 days before administration of study vaccine or planning to receive a COVID-19 vaccine during the first 14 days following study vaccination.

20	Planning to receive a vaccine during the first 28 days following the administration of study vaccine, other than COVID-19 vaccine.
21	Currently participating in another clinical study or planning to participate in another study during the study period, or administration of any investigational drug or medical device in the 4 weeks prior to study vaccination.
22	Prior receipt of blood, blood-derived products, or immunoglobulins in the 6 months prior to administration of study vaccine or planning to receipt such product during the study period.
23	Chronic administration (defined as 14 consecutive days in total) of immunosuppressants or other immune-modifying drugs during the period starting 90 days prior to vaccination or planned administration during the study (excluding topical, inhaled and intranasal preparations and intra-articular injections). For corticosteroids, this is prednisone >= 20 mg/day, or equivalent.
24	Current intake of more than 1 anticoagulant medication (coumarin derivatives, low molecular weight heparin, DOAC) or 1 anticoagulant medication in combination with antiaggregation medication.
25	Current anti-tuberculosis prophylaxis or therapy.
26	Elective surgery planned in the first 14 days following study vaccination.
27	WOCBP who are pregnant, breast-feeding or planning to become pregnant during the study.
28	Participants with history of any medical conditions that, in opinion of the investigator, might interfere with the results of the study or pose additional risk to the participants due to participation in the study.
29	Current febrile illness (oral temperature >38.0 °C) or other acute illness prior to vaccine administration. Participants with oral temperature >38.0 °C can be rescheduled to when they are at least 72 hours feverless as long as the delayed visit and randomization are still within the screening window defined by protocol.
30	Intake of antipyretics and/or analgesic medications within 24 hours prior to study vaccination. Reason for use (prophylaxis or treatment) should be documented. Participants can be rescheduled after being 24 hours free of intake of antipyretics and/or analgesic medications as long as the delayed visit and randomization are still within the screening window defined by protocol.

Supplementary Table 2. Protocol-defined Safety Laboratory tests.

Laboratory Tests	Parameters	
**Haematology**	• Platelet count	
	• RBC count	
	• RBC indices:	– Mean corpuscular volume (MCV)<br>– Mean corpuscular haemoglobin (MCH)
	• RBC	– % Reticulocytes
	• White blood cell (WBC) count with differential:	– Neutrophils<br>– Lymphocytes<br>– Monocytes<br>– Eosinophils<br>– Basophils
	• Haemoglobin	
	• Haematocrit	
**Biochemistry**	• Urea<br>• Creatinine<br>• C-reactive protein (CRP)<br>• High Sensitive C-reactive protein (CRP)(D0 and D1)	• Aspartate aminotransferase (AST)<br>• Alanine aminotransferase (ALT)<br>• Alkaline phosphatase<br>• Total and direct bilirubin
**Viremia (only on screening)**	• HBsAg	
	• HCV antibody	
	• HIV antibody	
**Pregnancy testing**	• Highly sensitive serum (**only screening**) and urine β-human chorionic gonadotropin (β-hCG) (**Vaccination visit day 0**) pregnancy test (as needed for WOCBP)	

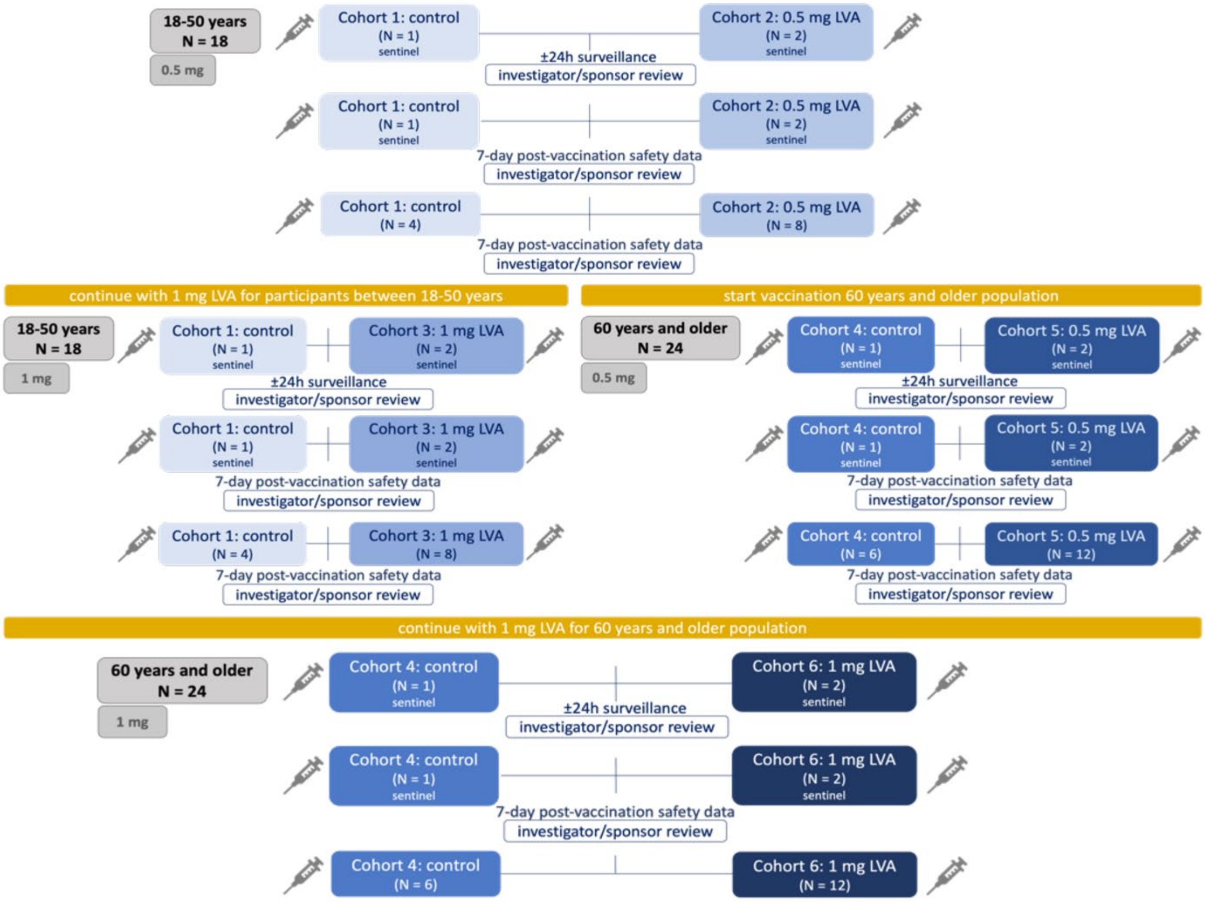

Supplementary Figure 1. Flowchart of the study design. A staggered study design, including sentinel participants, was implemented, starting with the low dose of LVA in younger adults and continuing to the high dose or the older adults after safety review by the investigators. Cohort 1 = YA, VaxigripTetra; Cohort 2 = YA, VaxigripTetra + LVA containing 0.5mg CMS; Cohort 3 = YA, VaxigripTetra + LVA containing 1mg CMS; Cohort 4 = OA, VaxigripTetra; Cohort 5 = OA, VaxigripTetra + LVA containing 0.5mg CMS; Cohort 6 = OA, VaxigripTetra + LVA containing 1mg CMS.

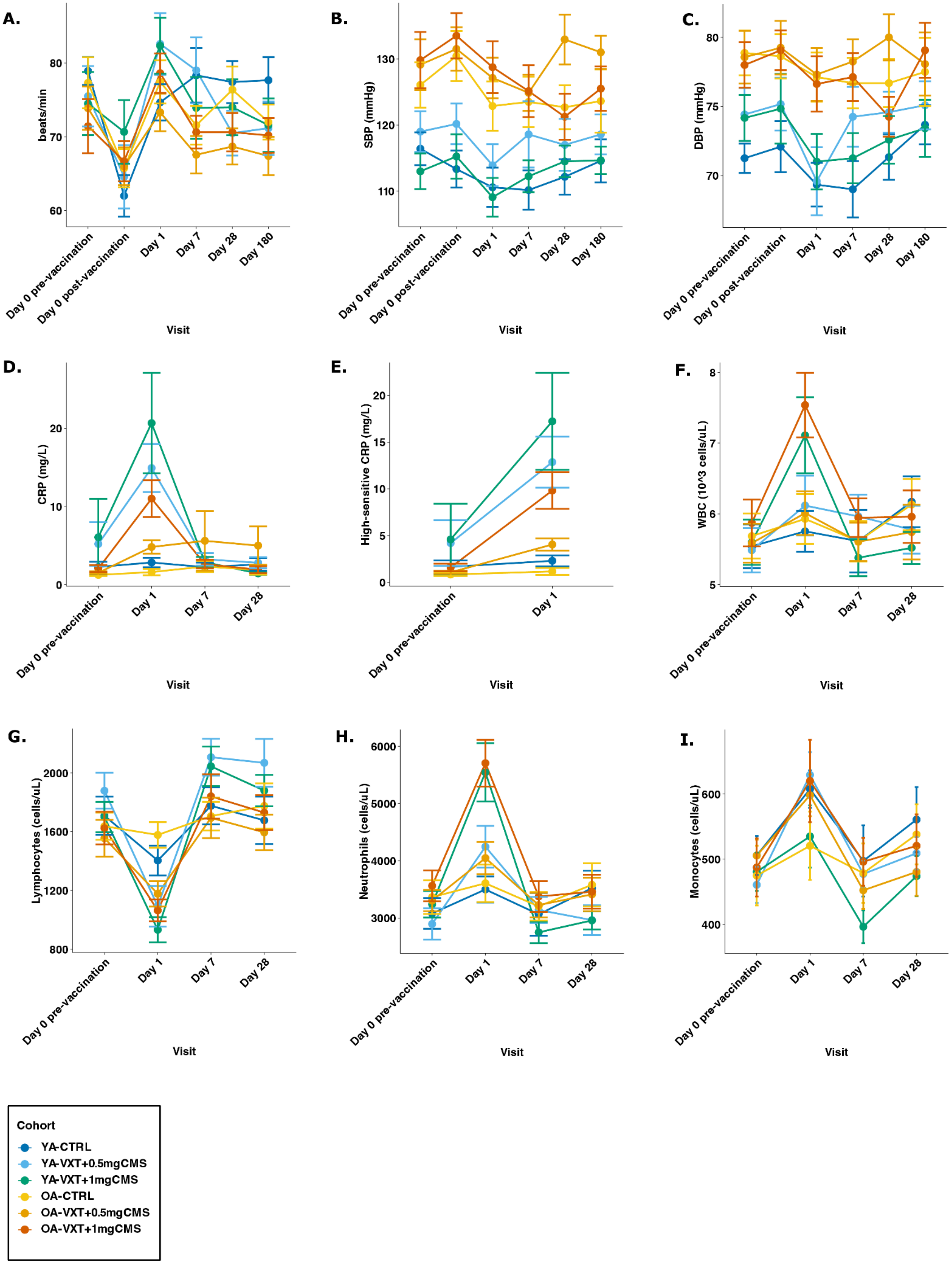
A.
beats/min
B.
SBP (mmHg)
C.
DBP (mmHg)
D.
CRP (mg/L)
E.
High-sensitive CRP (mg/L)
F.
WBC (10^3 cells/uL)
G.
Lymphocytes (cells/uL)
H.
Neutrophils (cells/uL)
I.
Monocytes (cells/uL)
Day 0 pre-vaccination
Day 0 post-vaccination
Day 1
Day 7
Day 28
Day 180
Visit
Cohort
YA-CTRL
YA-VXT+0.5mgCMS
YA-VXT+1mgCMS
OA-CTRL
OA-VXT+0.5mgCMS
OA-VXT+1mgCMS

Supplementary Figure 2. Vital signs and clinical safety laboratory parameters at baseline and at follow-up visits after vaccination for each cohort. A. Heart Rate (HR) in beats per min (bpm); B. Systolic Blood Pressure (SBP) in mmHg; B. Disatolic Blood Pressure (DBP) in mmHg; D. C-reactive protein (CRP) in mg/L; E. High-sensitive CRP in mg/L; F. White Blood Cell Count (WBC) in number x 10^3 cells/μL; G. Lymphocytes in number cells/μL; H. Nuetrophils in number cells/μL; I. Monocytes in number cells/μL; Dots represent the mean, bars represent standard error. YA = younger adults aged 18-50 years; OA = older adults 60 years or older. YA-CTRL= YA, VaxigripTetra; YA-VXT+0.5mgCMS = YA, VaxigripTetra + LVA containing 0.5mg CMS; YA-VXT+1mgCMS = YA, VaxigripTetra + LVA containing 1mg CMS; OA-CTRL = OA, VaxigripTetra; OA-VXT+0.5mgCMS = OA, VaxigripTetra + LVA containing 0.5mg CMS; OA-VXT+1mgCMS = OA, VaxigripTetra + LVA containing 1mg CMS.

Supplementary Table 3. Linear mixed-effects models of log2-transformed HI titres for each strain, incorporating Cohort, Visit, and their interaction, with random intercepts for subjects.

	**H3N2**			**H1N1**			**B/Austria**			**B/Phuket**		
Predictor	*Estimate*	*CI*	*p*	*Estimate*	*CI*	*p*	*Estimate*	*CI*	*p*	*Estimate*	*CI*	*p*
(Intercept)	4.45	3.3799 – 5.53	**<0.001**	4.99	3.99 – 5.99	**<0.001**	2.41	1.41 – 3.40	**<0.001**	3.95	3.00 – 4.90	**<0.001**
YA-VXT+0.5mgCMS	-0.33	-1.869 – 1.20	0.668	-0.08	-1.50 – 1.33	0.908	0.25	-1.15 – 1.65	0.726	-0.42	-1.76 – 0.92	0.541
YA-VXT+1mgCMS	0.92	-0.61 – 2.45	0.239	-1.38	-2.79 – 0.04	0.057	0.08	-1.32 – 1.49	0.907	-0.96	-2.30 – 0.38	0.161
OA-CTRL	-1.00	-2.43 – 0.43	0.170	-1.57	-2.90 – -0.25	**0.020**	0.79	-0.52 – 2.10	0.236	-1.28	-2.54 – -0.03	**0.045**
OA-VXT+0.5mgCMS	0.19	-1.24 – 1.62	0.797	-1.54	-2.87 – -0.22	**0.023**	1.26	-0.05 – 2.57	0.060	-1.09	-2.35 – 0.16	0.087
OA-VXT+1mgCMS	-0.84	-2.27 – 0.59	0.247	-0.60	-1.93 – 0.72	0.371	0.45	-0.86 – 1.76	0.502	-0.94	-2.19 – 0.32	0.142
Day 7	2.29	1.33 – 3.25	**<0.001**	3.21	2.13 – 4.29	**<0.001**	2.79	1.83 – 3.75	**<0.001**	2.46	1.62 – 3.30	**<0.001**
Day 28	3.21	2.25 – 4.17	**<0.001**	3.42	2.34 – 4.50	**<0.001**	3.33	2.37 – 4.29	**<0.001**	2.42	1.58 – 3.25	**<0.001**
Day 180	2.50	1.54 – 3.46	**<0.001**	2.46	1.38 – 3.54	**<0.001**	2.00	1.04 – 2.96	**<0.001**	1.67	0.83 – 2.50	**<0.001**
YA-VXT+0.5mgCMS × Day 7	1.88	0.52 – 3.23	**0.007**	0.67	-0.86 – 2.19	0.391	-0.21	-1.57 – 1.15	0.763	0.63	-0.56 – 1.81	0.300
YA-VXT+1mgCMS × Day 7	1.04	-0.32 – 2.40	0.133	2.08	0.56 – 3.61	**0.008**	0.33	-1.03 – 1.69	0.630	1.33	0.15 – 2.52	**0.028**
OA-CTRL × Day 7	0.55	-0.72 – 1.82	0.394	-0.18	-1.61 – 1.25	0.807	0.15	-1.13 – 1.42	0.822	-0.93	-2.04 – 0.18	0.101
OA-VXT+0.5mgCMS × Day 7	0.58	-0.69 – 1.85	0.367	-0.30	-1.73 – 1.13	0.678	1.05	-0.22 – 2.32	0.105	-0.43	-1.54 – 0.68	0.449

OA-VXT+1mgCMS × Day 7	1.55	0.28 – 2.82	**0.017**	0.51	-0.92 – 1.94	0.483	0.46	-0.81 – 1.73	0.479	-1.11	-2.22 – -0.01	**0.049**
YA-VXT+0.5mgCMS × Day 28	1.54	0.18 – 2.90	**0.026**	0.75	-0.78 – 2.28	0.335	-0.37	-1.73 – 0.98	0.588	1.04	-0.14 – 2.23	0.085
YA-VXT+1mgCMS × Day 28	0.13	-1.23 – 1.48	0.857	1.42	-0.11 – 2.94	0.069	-0.25	-1.61 – 1.11	0.718	1.13	-0.06 – 2.31	0.063
OA-CTRL × Day 28	0.26	-1.01 – 1.53	0.687	0.49	-0.94 – 1.92	0.501	0.26	-1.01 – 1.53	0.687	-0.54	-1.65 – 0.57	0.337
OA-VXT+0.5mgCMS × Day 28	0.14	-1.14 – 1.41	0.834	-0.26	-1.69 – 1.17	0.720	0.73	-0.54 – 2.00	0.260	0.02	-1.09 – 1.13	0.971
OA-VXT+1mgCMS × Day 28	1.57	0.30 – 2.84	**0.016**	0.71	-0.72 – 2.14	0.330	0.45	-0.82 – 1.72	0.489	-0.45	-1.56 – 0.66	0.427
YA-VXT+0.5mgCMS × Day 180	0.88	-0.48 – 2.23	0.206	0.50	-1.03 – 2.03	0.520	-0.75	-2.11 – 0.61	0.278	0.42	-0.77 – 1.60	0.490
YA-VXT+1mgCMS × Day 180	-0.12	-1.48 – 1.23	0.857	1.17	-0.36 – 2.69	0.134	-0.37	-1.73 – 0.98	0.588	0.50	-0.69 – 1.69	0.407
OA-CTRL × Day 180	-0.56	-1.83 – 0.71	0.385	-0.27	-1.70 – 1.16	0.709	0.44	-0.83 – 1.71	0.499	-0.92	-2.03 – 0.19	0.105
OA-VXT+0.5mgCMS × Day 180	-0.13	-1.41 – 1.15	0.843	-0.59	-2.04 – 0.85	0.417	0.81	-0.48 – 2.09	0.216	-0.15	-1.27 – 0.96	0.787
OA-VXT+1mgCMS × Day 180	1.47	0.20 – 2.74	**0.024**	0.39	-1.04 – 1.81	0.596	0.69	-0.58 – 1.96	0.288	-0.48	-1.59 – 0.63	0.396
**Random Effects**												

σ^2	1.43	1.81	1.43	1.09
τ_{00}	2.19 $_{Subject}$	1.31 $_{Subject}$	1.62 $_{Subject}$	1.70 $_{Subject}$
ICC	0.61	0.42	0.53	0.61
N	84 $_{Subject}$	84 $_{Subject}$	84 $_{Subject}$	84 $_{Subject}$
Observations	335	335	335	335
Marginal R^2 / Conditional R^2	0.427 / 0.774	0.505 / 0.713	0.445 / 0.739	0.376 / 0.756

CI: 95% Confidence Interval; p: p-value; YA = younger adults aged 18-50 years; OA = older adults 60 years or older; YA = younger adults aged 18-50 years; OA = older adults 60 years or older. YA-CTRL= YA, VaxigripTetra; YA-VXT+0.5mgCMS = YA, VaxigripTetra + LVA containing 0.5mg CMS; YA-VXT+1mgCMS = YA, VaxigripTetra + LVA containing 1mg CMS; OA-CTRL = OA, VaxigripTetra; OA-VXT+0.5mgCMS = OA, VaxigripTetra + LVA containing 0.5mg CMS; OA-VXT+1mgCMS = OA, VaxigripTetra + LVA containing 1mg CMS.

H3N2

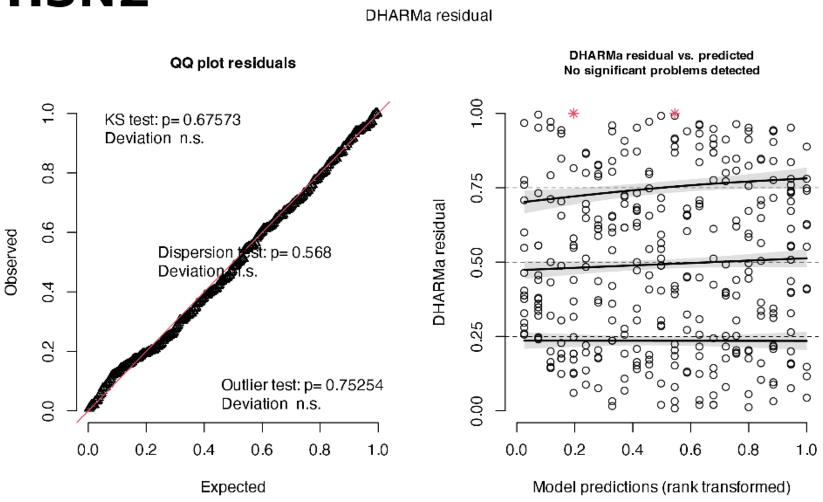

H1N1

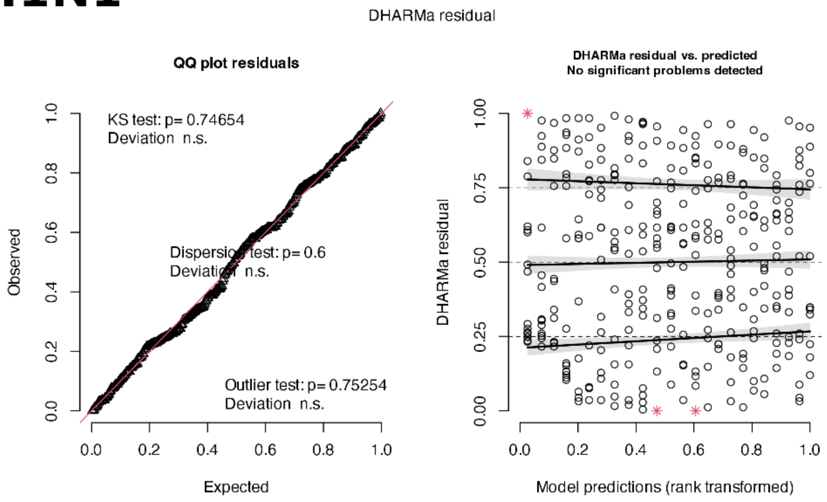

B/Austria

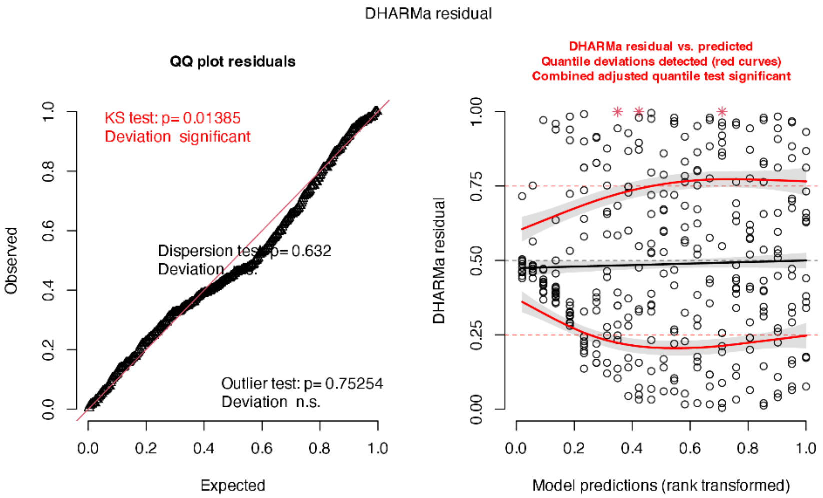

B/Phuket

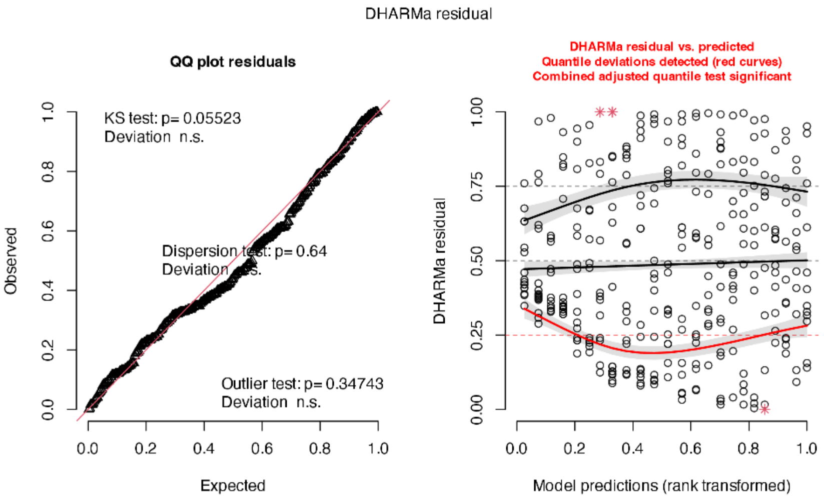

Figure S4: Diagnostic plots for LME models of HI; Figure ·

Supplementary Figure 3. Diagnostic plots from residual analysis for linear mixed-effects models assessing hemagglutination inhibition (HI) titres against four influenza strains: H3N2, H1N1, B/Austria, and B/Phuket. Each panel includes a QQ plot of residuals and residuals versus predicted values. KS tests for uniformity, outlier tests, and dispersion tests are reported. No significant deviations were observed for H3N2, H1N1, and B/Phuket. For B/Austria, the KS test indicated a significant deviation from uniformity ($p = 0.014$), and quantile deviations were detected, suggesting potential model misspecification. No significant outliers or dispersion issues were detected in any model.

Supplementary Table 4. Geometric microneutralization antibody titres (GMTs), per cohort per vaccine strain at baseline and 7, 28, and 180 days after vaccination.

			H3N2			H1N1			B/Austria			B/Phuket		
**YA**	Visit	Statistic	**CTRL**	**VXT+ 0.5mg CMS**	**VXT+ 1mg CMS**	**CTRL**	**VXT+ 0.5mg CMS**	**VXT+ 1mg CMS**	**CTRL**	**VXT+ 0.5mg CMS**	**VXT+ 1mg CMS**	**CTRL**	**VXT+ 0.5mg CMS**	**VXT+ 1mg CMS**
	Day 0	GMT	23.8	34.6	36.7	77.7	95.1	34.6	7.7	8.9	7.5	184.9	151.0	97.9
		95%CI	12.4 - 45.7	22.2 - 54.0	22.7 - 59.3	18.0 - 335.4	23.7 - 382.4	13.1 - 91.6	4.9 - 12.2	4.1 - 19.3	4.4 - 12.6	71.1 - 480.4	76.8 - 296.9	56.4 - 169.9
	Day 7	GMT	53.4	160.0	164.7	1317.5	2347.5	1974.0	49.0	53.4	41.2	1045.7	1522.2	1612.7
		95%CI	33.4 - 85.3	96.5 - 265.3	99.8 - 271.8	616.4 - 2816.3	1176.5 - 4684.0	676.4 - 5761.3	15.1 - 158.5	16.1 - 176.9	16.8 - 101.1	374.7 - 2918.1	791.6 - 2927.2	792.1 - 3283.4
		GMR	2.2	4.6	4.5	17.0	24.7	57.0	6.3	6.0	5.5	5.7	10.1	16.5
		95%CI	1.4 - 3.7	2.5 - 8.5	2.2 - 9.1	2.7 - 104.9	5.9 - 102.8	15.2 - 213.9	2.3 - 17.4	1.9 - 18.8	2.6 - 11.4	1.7 - 18.6	3.4 - 30.0	6.8 - 40.0
	Day 28	GMT	84.8	184.9	169.5	1660.0	2280.7	2635.0	67.3	109.9	82.3	1208.2	1478.9	1208.2
		95%CI	48.3 - 148.7	112.0 - 305.0	104.2 - 275.7	782.6 - 3520.8	972.9 - 5346.6	1106.2 - 6277.0	20.7 - 218.6	35.6 - 339.8	39.0 - 174.0	493.7 - 2956.4	749.2 - 2919.2	606.6 - 2406.1
		GMR	3.6	5.3	4.6	21.4	24.0	76.1	8.7	12.3	11.0	6.5	9.8	12.3
		95%CI	1.8 - 7.2	2.9 - 9.8	2.6 - 8.1	3.4 - 133.1	3.9 - 146.6	31.3 - 185.0	3.1 - 24.8	3.8 - 40.2	5.6 - 21.4	2.1 - 20.0	2.9 - 32.8	5.9 - 26.0
	Day 180	GMT	58.2	100.8	71.3	604.1	1076.3	931.6	40.0	61.7	51.9	553.9	553.9	479.5
		95%CI	30.7 - 110.4	65.3 - 155.5	47.2 - 107.7	341.7 - 1068.0	416.0 - 2784.8	336.7 - 2577.4	13.5 - 118.1	30.5 - 124.7	32.4 - 83.1	237.4 - 1292.7	280.6 - 1093.5	277.7 - 827.8
		GMR	2.4	2.9	1.9	7.8	11.3	26.9	5.2	6.9	6.9	3.0	3.7	4.9
		95%CI	1.3 - 4.5	1.7 - 4.8	1.3 - 3.0	1.9 - 31.0	1.9 - 68.5	10.7 - 67.7	1.9 - 14.4	3.1 - 15.2	4.1 - 11.8	1.4 - 6.5	1.4 - 9.9	2.6 - 9.3
**OA**	Visit	Statistic	**CTRL**	**VXT+ 0.5mg CMS**	**VXT+ 1mg CMS**	**CTRL**	**VXT+ 0.5mg CMS**	**VXT+ 1mg CMS**	**CTRL**	**VXT+ 0.5mg CMS**	**VXT+ 1mg CMS**	**CTRL**	**VXT+ 0.5mg CMS**	**VXT+ 1mg CMS**
	Day 0	GMT	19.5	28.9	10.4	16.6	24.3	47.6	14.8	23.3	13.0	57.9	64.4	101.5
		95%CI	13.9 - 27.6	14.9 - 56.1	6.6 - 16.4	8.9 - 31.1	10.4 - 56.9	17.1 - 132.0	7.4 - 29.7	12.2 - 44.3	7.7 - 21.9	39.3 - 85.3	37.7 - 110.0	62.5 - 164.9

Day 7	GMT	48.1	91.1	43.6	327.5	313.1	1030.7	115.8	241.5	186.2	179.6	320.0	415.0
	95%CI	28.0 - 82.8	43.5 - 190.6	20.4 - 93.4	107.6 - 996.8	116.1 - 844.6	442.9 - 2398.5	49.3 - 271.9	87.8 - 664.3	67.3 - 515.4	92.1 - 350.1	140.1 - 730.9	196.1 - 878.0
	GMR	2.5	3.2	4.2	19.7	12.9	21.7	7.8	10.4	14.4	3.1	5.0	4.1
	95%CI	1.6 - 3.9	1.8 - 5.6	2.1 - 8.2	7.9 - 49.1	6.2 - 27.0	8.9 - 52.7	3.7 - 16.6	3.4 - 32.0	6.4 - 32.2	1.7 - 5.6	2.7 - 9.1	1.9 - 8.6
Day 28	GMT	87.7	115.6	75.0	519.8	683.0	2152.7	211.1	397.4	349.0	272.2	482.9	599.7
	95%CI	38.0 - 202.9	60.9 - 219.6	35.2 - 159.4	213.9 - 1263.2	266.2 - 1752.2	1156.0 - 4008.6	97.9 - 455.2	189.9 - 831.5	149.3 - 815.6	135.3 - 547.7	196.3 - 1188.1	282.0 - 1275.5
	GMR	4.5	4.0	7.2	31.3	28.1	45.3	14.3	17.1	26.9	4.7	7.5	5.9
	95%CI	2.1 - 9.5	2.4 - 6.7	3.7 - 13.9	13.8 - 70.9	12.0 - 65.7	21.8 - 93.9	6.2 - 32.9	7.1 - 40.9	13.4 - 54.2	2.4 - 9.3	3.9 - 14.5	2.7 - 13.0
Day 180	GMT	33.2	48.1	43.6	145.9	206.3	668.3	115.8	206.3	149.9	142.5	298.6	334.2
	95%CI	20.6 - 53.8	26.2 - 88.2	23.6 - 80.5	72.3 - 294.1	85.0 - 500.8	380.4 - 1174.4	65.3 - 205.4	97.3 - 437.6	68.1 - 330.3	74.0 - 274.6	139.4 - 639.4	149.2 - 748.2
	GMR	1.7	2.0	4.2	8.8	7.6	14.1	7.8	8.2	11.6	2.5	4.5	3.3
	95%CI	1.2 - 2.4	1.4 - 2.7	2.5 - 7.0	4.3 - 17.9	3.4 - 17.0	6.4 - 30.8	3.6 - 16.8	3.8 - 17.5	6.3 - 21.3	1.3 - 4.7	2.7 - 7.6	1.5 - 7.3

GMT: geometric mean titre; GMR: geometric mean ratio compared to Day 0; 95%CI: 95% confidence interval. YA = younger adults aged 18-50 years; OA = older adults 60 years or older; CTRL = VaxigripTetra; VXT+0.5mgCMS = VaxigripTetra + LVA containing 0.5mg CMS; VXT+1mgCMS = VaxigripTetra + LVA containing 1mg CMS.

Supplementary Table 5. Linear mixed-effects models of log2-transformed MN titres for each strain, incorporating Cohort, Visit, and their interaction, with random intercepts for subjects.

	**H3N2**			**H1N1**			**B/Austria**			**B/Phuket**		
Predictors	*Estimate*	*CI*	*p*	*Estimate*	*CI*	*p*	*Estimate*	*CI*	*p*	*Estimate*	*CI*	*p*
(Intercept)	4.57	3.72 – 5.42	**<0.001**	6.28	5.00 – 7.56	**<0.001**	2.95	1.77 – 4.13	**<0.001**	7.53	6.51 – 8.55	**<0.001**
YA-VXT+0.5mgCMS	0.54	-0.66 – 1.74	0.375	0.29	-1.52 – 2.10	0.752	0.21	-1.46 – 1.88	0.806	-0.29	-1.74 – 1.15	0.692
YA-VXT+1mgCMS	0.62	-0.58 – 1.83	0.306	-1.17	-2.98 – 0.65	0.206	-0.04	-1.71 – 1.63	0.961	-0.92	-2.36 – 0.53	0.213
OA-CTRL	-0.34	-1.47 – 0.78	0.547	-2.15	-3.84 – -0.45	**0.013**	0.84	-0.72 – 2.41	0.288	-1.58	-2.94 – -0.23	**0.022**
OA-VXT+0.5mgCMS	0.28	-0.84 – 1.40	0.622	-1.68	-3.37 – 0.02	0.052	1.59	0.03 – 3.16	**0.045**	-1.52	-2.87 – -0.17	**0.028**
OA-VXT+1mgCMS	-1.19	-2.31 – -0.06	**0.038**	-0.71	-2.40 – 0.99	0.411	0.75	-0.81 – 2.31	0.345	-0.86	-2.22 – 0.49	0.210
Day 7	1.17	0.42 – 1.91	**0.002**	4.08	2.78 – 5.39	**<0.001**	2.67	1.59 – 3.74	**<0.001**	2.50	1.53 – 3.47	**<0.001**
Day 28	1.83	1.09 – 2.58	**<0.001**	4.42	3.11 – 5.72	**<0.001**	3.12	2.05 – 4.20	**<0.001**	2.71	1.74 – 3.68	**<0.001**
Day 180	1.29	0.55 – 2.04	**0.001**	2.96	1.66 – 4.26	**<0.001**	2.37	1.30 – 3.45	**<0.001**	1.58	0.61 – 2.55	**0.001**
YA-VXT+0.5mgCMS × Day 7	1.04	-0.01 – 2.09	0.052	0.54	-1.30 – 2.38	0.563	-0.08	-1.60 – 1.43	0.914	0.83	-0.54 – 2.20	0.233
YA-VXT+1mgCMS × Day 7	1.00	-0.05 – 2.05	0.062	1.75	-0.09 – 3.59	0.062	-0.21	-1.73 – 1.31	0.787	1.54	0.17 – 2.91	**0.028**
OA-CTRL × Day 7	0.11	-0.87 – 1.10	0.819	0.10	-1.62 – 1.83	0.905	0.18	-1.24 – 1.60	0.806	-0.97	-2.25 – 0.31	0.138
OA-VXT+0.5mgCMS × Day 7	0.49	-0.49 – 1.47	0.329	-0.40	-2.12 – 1.33	0.651	0.71	-0.71 – 2.13	0.327	-0.19	-1.47 – 1.10	0.774

OA-VXT+1mgCMS × Day 7	0.90	-0.09 – 1.88	0.074	0.35	-1.37 – 2.08	0.686	1.18	-0.24 – 2.60	0.104	-0.47	-1.75 – 0.81	0.473
YA-VXT+0.5mgCMS × Day 28	0.58	-0.47 – 1.64	0.276	0.17	-1.67 – 2.01	0.859	0.50	-1.02 – 2.02	0.517	0.58	-0.79 – 1.95	0.403
YA-VXT+1mgCMS × Day 28	0.38	-0.68 – 1.43	0.484	1.83	-0.01 – 3.67	0.051	0.33	-1.18 – 1.85	0.666	0.92	-0.45 – 2.29	0.189
OA-CTRL × Day 28	0.32	-0.66 – 1.31	0.519	0.58	-1.14 – 2.31	0.506	0.66	-0.76 – 2.08	0.364	-0.61	-1.90 – 0.67	0.347
OA-VXT+0.5mgCMS × Day 28	0.17	-0.82 – 1.15	0.739	0.40	-1.33 – 2.12	0.651	0.97	-0.45 – 2.39	0.180	0.20	-1.08 – 1.48	0.762
OA-VXT+1mgCMS × Day 28	1.01	0.03 – 1.99	**0.044**	1.08	-0.64 – 2.81	0.217	1.63	0.21 – 3.04	**0.025**	-0.15	-1.43 – 1.14	0.823
YA-VXT+0.5mgCMS × Day 180	0.25	-0.80 – 1.30	0.641	0.54	-1.30 – 2.38	0.563	0.42	-1.10 – 1.93	0.589	0.29	-1.08 – 1.66	0.676
YA-VXT+1mgCMS × Day 180	-0.33	-1.39 – 0.72	0.534	1.79	-0.05 – 3.63	0.056	0.42	-1.10 – 1.93	0.589	0.71	-0.66 – 2.08	0.310
OA-CTRL × Day 180	-0.51	-1.49 – 0.47	0.308	0.35	-1.37 – 2.08	0.686	0.59	-0.83 – 2.01	0.411	-0.36	-1.65 – 0.92	0.576
OA-VXT+0.5mgCMS × Day 180	-0.40	-1.39 – 0.60	0.434	0.04	-1.70 – 1.78	0.964	0.82	-0.61 – 2.26	0.258	0.57	-0.72 – 1.87	0.385
OA-VXT+1mgCMS × Day 180	0.77	-0.21 – 1.76	0.124	0.85	-0.87 – 2.58	0.330	1.16	-0.26 – 2.58	0.110	0.14	-1.15 – 1.42	0.836
**Random Effects**												

σ^2	0.86	2.63	1.78	1.46
τ_{00}	1.37 $_{Subject}$	2.46 $_{Subject}$	2.53 $_{Subject}$	1.78 $_{Subject}$
ICC	0.62	0.48	0.59	0.55
N	84 $_{Subject}$	84 $_{Subject}$	84 $_{Subject}$	84 $_{Subject}$
Observations	335	335	335	335
Marginal R^2 / Conditional R^2	0.328 / 0.742	0.490 / 0.737	0.403 / 0.753	0.378 / 0.720

CI: 95% Confidence Interval; p: p-value; YA = younger adults aged 18-50 years; OA = older adults 60 years or older. YA-CTRL= YA, VaxigripTetra; YA-VXT+0.5mgCMS = YA, VaxigripTetra + LVA containing 0.5mg CMS; YA-VXT+1mgCMS = YA, VaxigripTetra + LVA containing 1mg CMS; OA-CTRL = OA, VaxigripTetra; OA-VXT+0.5mgCMS = OA, VaxigripTetra + LVA containing 0.5mg CMS; OA-VXT+1mgCMS = OA, VaxigripTetra + LVA containing 1mg CMS.

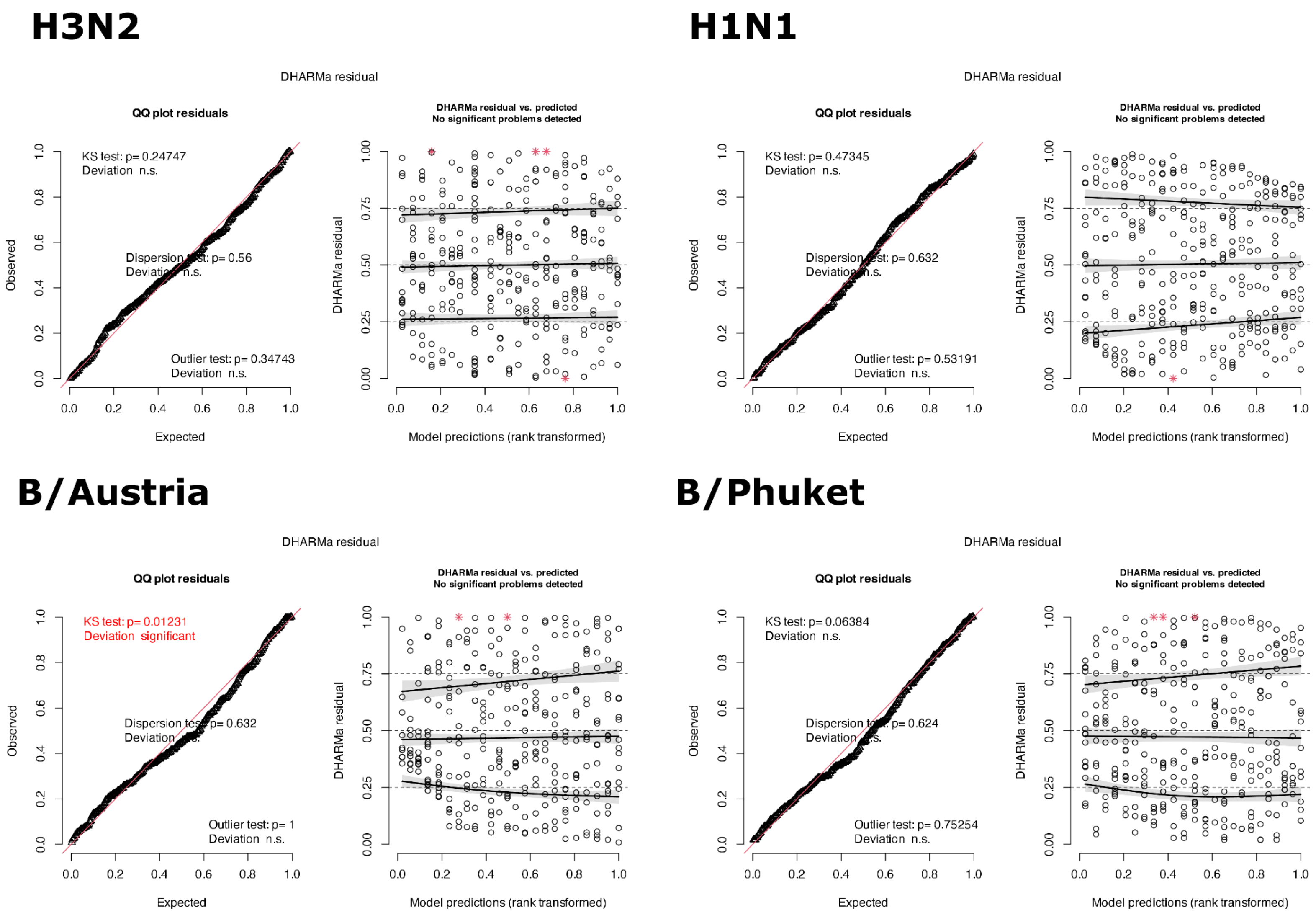

Supplementary Figure 4. Diagnostic plots from residual analysis for linear mixed-effects models assessing hemagglutination inhibition (HI) titres against four influenza strains: H3N2, H1N1, B/Austria, and B/Phuket. Each panel includes a QQ plot of residuals and residuals versus

predicted values. KS tests for uniformity, outlier tests, and dispersion tests are reported. No significant deviations were observed for H3N2, H1N1, and B/Phuket. For B/Austria, the KS test indicated a significant deviation from uniformity ($p = 0.012$), suggesting potential model misspecification. No significant outliers or dispersion issues were detected in any model.
